# The impacts of the COVID-19 pandemic on indirect costs of mental illness and behavioral disorders in Poland

**DOI:** 10.3389/fpubh.2023.1207389

**Published:** 2023-09-18

**Authors:** Karolina Sobczyk, Tomasz Holecki, Anna Rogalska

**Affiliations:** Department of Economics and Health Care Management, Faculty of Public Health in Bytom, Medical University of Silesia in Katowice, Katowice, Poland

**Keywords:** indirect costs, mental illness, behavioral disorders, absenteeism, COVID-19

## Abstract

**Introduction:**

In various research, the estimation of the disease’s economic burden has been taken into consideration. But given the fact that different settings will have distinguished consequences, determining the economic burden of COVID-19 in the studied environment is of great importance. As a result, this study aimed to show the change in indirect costs of mental health problems due to the COVID-19 pandemic in Poland.

**Methods and Results:**

Indirect costs related to mental health problems were analyzed from the perspective of the Social Insurance Institution in Poland. In 2021, they amounted to about 285.8 billion PLN (Polish currency) [61.1 billion EUR (European currency)], up 6% from the previous year. A large increase in spending on disability benefits was observed for 2019–2021 (+14.7%). Disease groups generating the highest expenditures in the structure of total expenditures on incapacity benefits in 2021 in Poland were mental health problems (16.7% of total expenditures). Expenditures on disability benefits related to mental health problems incurred by Social Security in 2021 amounted to about 7.42 billion PLN [1.6 billion EUR] and were 19.4% higher than in 2019 (before the pandemic). In the 2012–2019 period, there was a significant decrease in expenses related to inpatient rehabilitation (41.3%), while in 2020–2021, these expenses decreased several times as the epidemiological situation related to the COVID pandemic reduced access to such services.

**Discussion:**

This is the first study on the economic burden of COVID-19 indirect costs in Poland. Calculating the economic impact is crucial, particularly when there is a large disease outbreak and countries are severely constrained by financial resources. Doing so could aid in the development of effective social security policies. As shown in this study, the indirect costs of absenteeism expenses due to mental health problems increased significantly during the COVID-19 pandemic. It is necessary to take all possible measures, both in the field of primary and secondary prevention, to prevent disability and exclusion from the labor market of people affected by mental health problems, which is justified by epidemiological data and financial data on the expenses incurred by Social Security for social insurance benefits.

## 1. Introduction

Globally, there have already been a very large number of cases of the new coronavirus disease (COVID-19), which is caused by the SARS-CoV-2 virus. By April 6, 2023, this fatal disease has infected more than 762201169 million people and nearly 6893190 million have died as a result of the disease (1). Global health systems are facing significant difficulties in avoiding infections, recognizing and managing COVID-19 cases, and providing efficient public health protection measures as a result of COVID-19’s rapid spread (2, 3). Although these difficulties largely result from an infectious condition with implications for physical health, they may also have a significant negative impact on mental health and well-being (4, 5). People all around the world struggle with anxiety and dread about their safety, the lack of a viable vaccine or cure, and negative socioeconomic effects including unemployment and restricted access to necessities as a result of lockdown and quarantine measures in various situations (6–9). Researchers and practitioners in global health must pay attention to these challenges because they may have various effects on mental health across populations. Previous research indicates that significant economic crises or natural disasters are frequently followed by depression, anxiety disorders, substance addiction, increased suicidal thoughts, and PTSD (10–12). It is stressed that the COVID-19 pandemic has had a huge impact on the mental health of the general public, and workers in particular, and in this context the transition to remote work has had the greatest impact (13–15).

Another issue with global health is the effect COVID-19 has on those who test positive psychologically (16). This vulnerable demographic is affected by several issues, including social isolation following a diagnosis of the illness, stigma and prejudice, lengthy hospitalization, and a lack of social support (7, 17, 18). These difficulties could become more common in COVID-19 along with psychological pressures that affect people generally. In addition, individuals with preexisting diseases or those with poor access to healthcare are more likely to experience psychological stress during this epidemic (19, 20). Furthermore, individuals and populations may have experienced several mental health issues before the start of the pandemic, which could make them more vulnerable to negative mental health outcomes after receiving a COVID-19 diagnosis (21, 22). More and more studies and reports indicate that COVID-19 individuals may experience depression, anxiety disorders, psychological discomfort, and suicidal conduct, which calls for a thorough study of the pandemic’s mental health epidemiology (18, 23, 24).

Diseases, such as COVID-19, bring not only direct medical costs such as hospitalization, medicine, and doctor consultations but also indirect costs that are not as easily quantifiable. These indirect costs can have a lasting impact on individuals, families, communities, and society as a whole. One of the major indirect costs of diseases is loss of productivity (25). When someone falls ill, they may miss work and lose income or be less productive at work due to reduced energy levels and concentration. This not only affects the individual but also their family who may have to make sacrifices to make up for the loss of income (26).

Another indirect cost of diseases is the burden placed on caregivers. Family members or friends may have to take time off work to care for the sick person, and in some cases, may have to give up their careers to become full-time caregivers. This can lead to financial strain and emotional stress on the caregivers as well as the patient. Diseases can also have a ripple effect on the economy (27). When a large number of people fall ill, it can lead to reduced economic activity and lower GDP (28). The cost of healthcare also rises, which puts a strain on government budgets and can lead to cuts in other areas such as education and infrastructure. Furthermore, diseases can also have long-term effects on people’s lives such as disabilities, reduced quality of life, and premature death. These consequences can lead to a loss of potential economic productivity and put an additional burden on social welfare programs (15, 29).

In various research, the estimation of the disease’s economic burden has been taken into consideration (26, 30, 31). But given the fact that different settings will have distinguished consequences, determining the economic burden of COVID-19 in the studied environment is of great importance. As a result, this study aimed to show the change in indirect costs of mental illness and behavioral disorders due to the COVID-19 pandemic.

## 2. Materials and methods

### 2.1. Study design and settings

The study is a cost analysis. Indirect costs related to mental illness and behavioral disorders were analyzed from the perspective of the Social Insurance Institution in Poland. All cost values were presented in PLN (Polish currency). According to the National Bank of Poland, the euro exchange rate on 11/04/2023 was PLN 4.68. For ease of reference, conversions to EUR (European currency) are shown in parentheses.

### 2.2. Data collection

Disability cost data were extracted based on the International Statistical Classification of Diseases and Health Problems – Tenth Revision (ICD-10) codes: F00-F99 “Mental and behavioral disorders.” The analysis of indirect costs was based on a retrospective evaluation of Social Insurance Institution data for 2012–2021, which was provided by this public finance sector institution.

In the Classification of Mental Disorders (DSM-5), behavioral disorders are defined as those that do not fit into accepted social norms and standards set by law, e.g., refusal to go to school, aggression toward others, and destruction of objects.^1^

The study did not require the approval of a bioethics committee under current Polish legislation.

The research was conducted using Statistica 13.0 software. Mann–Whitney U and Kruskal-Wallis statistical tests were used in the statistical processing of the data. The probability level was set at *p* = 0.05.

## 3. Results

### 3.1. Expenditure on social security benefits related to incapacity due to mental illness and behavioral disorders

The amount of expenditures on cash benefits realized by the Social Insurance Institution has shown an upward trend over the past decade. In 2021, they amounted to about 285.8 billion PLN [61.1 billion EUR] up 6% from the previous year. Since 2016, the main item of expenditures on incapacity benefits has been sickness absence and disability benefits – in 2021 this was 52.2 and 28% of total expenditures, respectively. The structure of the discussed group of expenditures in 2021 also included expenditures on social pensions (8.4%), rehabilitation benefits (5.2%), and therapeutic rehabilitation under Social Security disability prevention (0.2%) (32).

**Figure 1:**
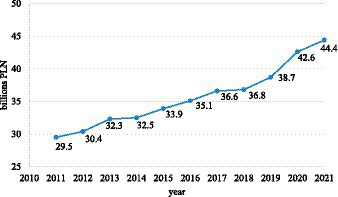
Total Social Security disability benefit expenditures incurred by the Social Insurance Institution in 2011–2020 (in billion zlotys). Own compilation based on: Social Insurance Institution, Expenses for social insurance benefits related to inability to work in 2011–2021.

Total Social Security disability benefit expenditures incurred by the Social Insurance Institution in 2021 amounted to more than 44.4 billion PLN [9.5 billion EUR], increasing by 14.8 billion PLN [3.2 billion EUR], compared to 2011. The amount of these expenditures in the year under review accounted for 1.7% of GDP, and this share decreased by 0.1 percentage points compared to the previous year. The observed large increase in spending on disability benefit expenditures in 2019–2021 (+14.7%) is due to the occurrence of the COVID-19 pandemic. The detailed data is illustrated using Figure 1.

Disease groups generating the highest expenditures in the structure of total expenditures on incapacity benefits in 2021 in Poland were mental disorders and behavioral disorders (16.7% of total expenditures), diseases of the osteoarticular, muscular, and connective tissue systems (14.5%), diseases related to pregnancy, childbirth and the postpartum period (12.5%), injuries, poisoning and other specified effects of external agents (12.2%), diseases of the respiratory system (8.2%), the circulatory system (8%) and the nervous system (7.7%). All of the aforementioned disease groups accounted for about 80% of incapacity expenses (32).

Expenditures on disability benefits related to mental and behavioral disorders incurred by Social Security in 2021 amounted to about 7.42 billion PLN [1.6 billion EUR] and were 19.4% higher than in 2019 (before the pandemic) and 41.2% higher than in 2012. For men, these expenditures have increased by 33.3% over the past 10 years, (from 2.7 billion PLN to 3.6 billion PLN [from 0.6 billion EUR to 0.8 billion EUR]) for women – by 50% (from 2.4 billion PLN to 3.6 billion PLN [from 0.5 billion EUR to 0.8 billion EUR]). It is also noteworthy that while in the case of men the share of expenditures related to incapacity due to mental and behavioral disorders in the structure of total expenditures increased by 1 percentage point, a decreasing trend was observed for women (a decrease of 0.7 percent points). Detailed data are shown in Table 1.

**Table 1:** Expenditures for benefits related to incapacity due to mental and behavioral disorders incurred by the Social Insurance Institution from 2012 to 2021 by gender in billion zlotys and as a % of the total in the structure of expenditures.

Gender	Total		Men		Women	
Year	Billions PLN [EUR]	%	Billions PLN [EUR]	%	Billions PLN [EUR]	%
2012	5.1 [1.1]	16.7	2.7 [0.6]	15.8	2.4 [0.5]	17.7
2013	5.4 [1.2]	16.8	2.9 [0.6]	16.0	2.5 [0.5]	17.7
2014	5.6 [1.2]	17.3	3.0 [0.6]	16.9	2.6 [0.6]	17.9
2015	5.9 [1.3]	17.4	3.1 [0.7]	17.2	2.8 [0.6]	17.7
2016	5.6 [1.2]	16.0	2.9 [0.6]	15.6	2.7 [0.6]	16.5
2017	5.8 [1.2]	15.9	3.0 [0.6]	15.7	2.8 [0.6]	16.1
2018	5.8 [1.2]	15.8	3.0 [0.6]	15.8	2.8 [0.6]	15.8
2019	6.2 [1.3]	16.2	3.2 [0.7]	16.3	3.0 [0.6]	16.1
2020	7.2 [1.5]	17.1	3.6 [0.8]	16.8	3.6 [0.8]	17.3
2021	7.4 [1.6]	16.7	3.7 [0.8]	16.5	3.7 [0.8]	17.0

Own compilation based on: Social Security, Expenditures on social insurance benefits related to inability to work in 2012–2021.

In assessing the level of spending on incapacity-related benefits, the rate of spending per person covered by health insurance is also important. Mental and behavioral disorders, unchanged for the past 10 years, rank first in the ranking of disease groups generating the highest average spending per person (about 295 PLN in 2020 [63 EUR]). These expenses were 15.6% higher than in 2019 (about 256 PLN [54.7 EUR]) and 33.8% higher than in 2012 (about 222 PLN [47.4 EUR]) (33). The fact that mental and behavioral disorders are a significant burden on the social security system is also evidenced by the fact that among the 20 disease entities generating the highest expenditures on incapacity benefits in 2021 were as many as six from the group in question. These are schizophrenia, stress reaction and adaptive disorders, moderate intellectual disability, depressive episode, anxiety disorders other than a phobia, and recurrent depressive disorder, generating a total of 11.5% of total expenditures on incapacity benefits (32).

The difference between the periods depends on the number of patients admitted to the wards due to the prevailing COVID-19 pandemic, especially in its first phases (the year 2020) (*p* < 0.05).

### 3.2. Costs of short-term absenteeism due to mental and behavioral disorders

In 2021, a total of 23.1 million medical certificates for 252 million days were issued to people insured with Social Security. Of this number, 20.5 million certificates were certificates issued for self-inflicted illness. The number of days of sickness absence from these certificates was 239.9 million days (57.2% were for women, 42.8% for men), and the average length of the certificate was 11.73 days. Mental and behavioral disorders ranked fifth among the most common causes of sickness absence, accounting for 25.2 million days of absence from work (10.5% of the total) (34).

Mental and behavioral disorders, as already mentioned, occupy the first place in the structure of expenses incurred by Social Security for the payment of benefits related to the inability to work in total (16.7% in 2021). Restricting the analysis of the data solely to expenses incurred for sickness absence – they occupy the 3rd place (11.4%), after diseases of pregnancy, childbirth, and the puerperium (22.6%) and diseases of the osteoarticular, muscular, and connective tissue systems (13.7%). Noteworthy is the fact that limiting the analysis of expenditures only to those incurred in connection with sickness absence for men puts diagnoses in the group of mental and behavioral disorders in fourth place (11.3% of the total in 2020). For women, it ranks second (11.5% of the total in 2021), after diseases caused by pregnancy, childbirth, and puerperium (36.9%). As for rehabilitation benefits, mental disorders and behavioral disorders rank second among the disease groups generating the highest costs associated with their payment (17.9% of the total in 2021), after diseases of the osteoarticular, muscular, and connective tissue systems (31.2%) (32).

Under Social Security disability prevention, dysfunctions resulting from diseases of the musculoskeletal system, cardiovascular system, respiratory system, psychosomatic diseases, vocal system, and oncological diseases are subject to medical rehabilitation. Expenses related to conditions occurring in the group of mental and behavioral disorders, do not dominate the structure of expenses for inpatient rehabilitation (3.3% of the total in 2021). Much higher costs are generated in this case by diseases of the musculoskeletal system (58.9%), injuries and poisonings (10.7%), diseases of the nervous system (10%), the cardiovascular system (6.5%), or the respiratory system (5.9%) (32). Between 2012 and 2021, there was a significant increase in expenses incurred by Social Security for short-term absenteeism for sickness benefits and rehabilitation benefits. In the 2012–2019 period, there was a significant decrease in expenses related to inpatient rehabilitation (41.3%), while in 2020–2021, these expenses decreased several times as the epidemiological situation related to the COVID pandemic reduced access to such services (33). Expenditures related to sickness absence in 2021 increased by about 41.8%, compared to 2019 (before the pandemic). For rehabilitation benefits, this was an increase of 53.5% over the same period. Detailed data on Social Security’s expenditures on benefits related to incapacity for work due to mental and behavioral disorders in 2012–2021 due to short-term absenteeism are presented in Table 2.

**Table 2:** Expenditures on benefits related to incapacity for work due to mental and behavioral disorders incurred by Social Security from 2012 to 2020 in connection with short-term absenteeism in millions of zlotys and as a % of the total in the structure of expenditures.

Benefit	Sickness absence		Rehabilitation benefits		Disability prevention	
Year	Billions PLN [EUR]	%	Billions PLN [EUR]	%	Billions PLN [EUR]	%
2012	907.3 [193.9]	7.4	136.2 [29.1]	12.2	12.1 [2.6]	7.4
2013	1070.4 [228.7]	8.0	169.8 [36.3]	13.8	12.1 [2.6]	7.2
2014	1139.6 [243.5]	8.4	180.7 [38.6]	14.1	13.5 [2.9]	8.0
2015	1359.8 [290.6]	9.0	221.5 [47.3]	16.2	14.1 [3.0]	8.3
2016	1535.9 [328.2]	9.4	241.7 [51.6]	15.8	11.9 [2.5]	6.8
2017	1662.5 [355.2]	9.4	256.0 [54.7]	15.4	11.1 [2.4]	6.1
2018	1734.9 [370.7]	9.4	264.5 [51.6]	15.5	7.8 [1.7]	4.1
2019	1977.2 [422.5]	10.0	269.7 [54.7]	14.5	7.1 [1.5]	3.5
2020	2680.8 [572.8]	11.7	383.8 [56.5]	17.0	2.4 [0.5]	3.8
2021	2803.5 [599.0]	11.4	414.1 [57.6]	17.9	3.1 [0.7]	3.3

Own compilation based on: Social Insurance Institution, Expenditures on social insurance benefits related to inability to work in 2012–2021.

Based on the analysis, the cost of mental disorders since the COVID-19 pandemic has increased compared to previous years (T = 10.654; r = 0.531; *p* = 0.001).

### 3.3. Costs of long-term absenteeism due to mental and behavioral disorders

For incapacity pensions, the highest expenses in 2021 were for injuries and poisoning (17.1%), cardiovascular diseases (16.6%), osteoarticular diseases (16.3%), and mental and behavioral disorders (15%). The highest share of expenditures, in the case of mental and behavioral disorders, is recorded for partial disability pensions (46.1% of pension expenditures in this disease group) and total disability pensions (37.6%); the remainder is for total disability and independent living pensions (16.3%) (32). From 2012 to 2021, there was a significant decrease in the expenses incurred by Social Security for disability pensions due to mental and behavioral disorders (29.5%) (33). The 2019–2021 period also saw a slight decrease in this area of spending (about 4%). Detailed data on Social Security’s expenditures on disability benefits due to mental and behavioral disorders in 2012–2021 in connection with long-term absenteeism are presented in Table 3.

**Table 3:** Expenditures for disability benefits related to mental and behavioral disorders, incurred by Social Security from 2012 to 2021 in connection with long-term absenteeism in millions of zlotys and as a % of the total in the structure of expenditures.

Benefit	Disability pensions	
Year	Billions PLN [EUR]	%
2012	2922.8 [624.5]	19.4
2013	2998.5 [640.7]	19.2
2014	3118.9 [666.4]	20.0
2015	3207.5 [685.4]	20.9
2016	2591.4 [553.7]	17.3
2017	2417.4 [516.5]	16.5
2018	2201.9 [470.5]	16.1
2019	2142.6 [457.8]	15.8
2020	2135.6 [456.3]	15.6
2021	2059.6 [440.1]	15.0

Own compilation based on: Social Insurance Institution, Expenditures on social insurance benefits related to inability to work in 2012–2021.

Based on the analysis, the cost of mental disorders since the COVID-19 pandemic has increased compared to previous years (T = 11.965; r = 0.674; *p* = 0.002).

## 4. Discussion

This is the first study on the economic burden of COVID-19 indirect costs in Poland. Calculating the economic impact is crucial, particularly when there is a large disease outbreak and countries are severely constrained by financial resources. Doing so could aid in the development of effective social security policies.

The social cost of COVID-19 far transcends the medical costs. Marziyeh et al. study on COVID-19 costs showed that only productivity losses due to premature death per patient were $83410.82 that in comparison with average direct medical costs is 58 times (25). The average indirect cost of premature death is very similar to the amount determined in another Iranian study (31). When the productivity lost as a result of hospitalization and the subsequent healing process is taken into account, the societal costs in Marziyeh et al. (25) study will exceed this sum. According to the findings of this study, the economic burden of COVID-19 in Bushehr province in Iran is estimated to be $43.97 million ($39.47 and $205.20 million) and 32% of this constitutes direct medical costs. In other words, the share of societal or indirect costs is more than twofold.

A large share of indirect costs in the economic burden of COVID-19 is seen among medical personnel. Healthcare workers (HCP) have been identified as a high-risk group for SARS-CoV-2 infection from the very beginning of the epidemic, and elevated absence rates and shortages of HCP have been observed (35–40). A recent systemic review of 594 sources found a total of 152,888 reported infections and 1413 deaths among HCP during the first pandemic wave worldwide (36). In addition to the safety issues with HCP, exposure and infection of front-line HCP necessitate the allocation of financial resources for their monitoring and care, which worsens the scarcity of HCP due to elevated absenteeism rates (38). In Maltezou et al. (30) study, absenteeism was the primary factor in both categories of HCPs’ overall expenditures. The significant negative effects of absence are in part attributable to its protracted duration, whether it be for isolation needs (healthy absenteeism) in compliance with Greek national guidelines (35) or in the setting of symptomatic disease (COVID-19). For a mean period of 7.5 days, absenteeism was recorded in 40% of exposed HCP, and for a mean period of 25.8 days in 99% of HCP with COVID-19. Results from Maltezou et al. (30) were consistent with a study from Spain where 65 symptomatic workers (24.6%) at a long-term care facility missed a mean of 19.2 days of work during an epidemic of COVID-19 (41).

The consequences of mental illnesses and behavioral disorders, especially those of a chronic nature, significantly affect the reduction of the psycho-physical performance of individuals, including the ability to engage in employment and limitations in the performance of activities of daily living. The phenomenon of sickness absence, both short-term and long-term, affects many aspects of society’s functioning, is a measure of the population’s health situation, and is an important indirect cost of illness. The scale of absenteeism indicates, among other things, the effectiveness of the healthcare system and the labor market situation (42). As indicated in the publication, mental illnesses and behavioral disorders rank first in the ranking of conditions that generate the highest costs of lost productivity borne by the social security system. They also rank first in the ranking of disease groups generating the highest average expenditures per insured person. Significantly, the last decade has seen a significant increase in all expenses incurred by Social Security for short-term absenteeism due to the group of conditions in question.

## 5. Strengths and limitations

An important achievement of the study is the collection in one place of all the necessary data on the cost of mental disorders, taking into account the predictive factor that was the COVID-19 pandemic. The conclusions of the study can be used in the wide-ranging health promotion and prevention and future psychoeducation of the public. Being guided by the results of the study can help in designing such undertakings as health policy programs or national health strategies. In addition, the study is not free of limitations. First of all, the data is based on reports that are available electronically, and it is worthwhile to include in future research the raw data collected by individual centers. It should also be emphasized that the results cannot be generalized and show only the national state.

## 6. Conclusion

As shown in this study, the indirect costs of absenteeism expenses due to mental illness and behavioral disorders increased significantly during the COVID-19 pandemic. The COVID-19 epidemic drastically changed how people behave, work, and interact with one another in a very short amount of time. Never before has society depended so heavily on modern technology and the internet for communication and production, and many of these changes are likely to persist far beyond the current public health crisis. Additionally, the pandemic has provided us with two crucial but extremely tough lessons regarding mental health. The first is that those with mental illnesses are disproportionately affected by such events. The additional travel limitations, social isolation, and house confinement — all necessary measures to contain the epidemic — make them vulnerable in addition to going against what is often utilized in cognitive and behavioral therapy to effectively treat these problems. The majority of therapeutic advancement must be made by patients when they are not with their doctor, according to a second lesson. Patients need to keep in mind to remember to take their prescriptions, avoid risk factors, and engage in adaptive behaviors or exercises during those times, which are frequently spent at home and alone. During confinement, many people with mental illnesses were unprepared for such autonomy and self-help, and society was unprepared to assist them. For these reasons, the viral pandemic that is COVID-19 has also highlighted the existence of a chronic and major mental health crisis (43).

It is necessary to take all possible measures, both in the field of primary and secondary prevention, to prevent disability and exclusion from the labor market of people affected by mental and behavioral disorders, which is justified by epidemiological data and financial data on the expenses incurred by Social Security for social insurance benefits. A reliable assessment of the number of public funds allocated to finance the indirect costs of diseases should be an integral part of prioritizing investments in specific areas of health. These decisions should also take into account epidemiological data and projections on morbidity and prevalence. Only properly made decisions that improve the economic efficiency and clinical effectiveness of health care will lead to improvements in the health of individuals and society as a whole.

## Data availability statement

The original contributions presented in the study are included in the article/supplementary material, further inquiries can be directed to the corresponding author.

## Ethics statement

Ethical review and approval was not required for the study on human participants in accordance with the local legislation and institutional requirements. Written informed consent from the patients/participants or patients/participants’ legal guardian/next of kin was not required to participate in this study in accordance with the national legislation and the institutional requirements.

## Author contributions

KS and TH: conceptualization, writing—original draft preparation, writing—review, and editing. KS: methodology, resources, and supervision. KS and AR: formal analysis. AR: investigation. All authors contributed to the article and approved the submitted version.

## Conflict of interest

The authors declare that the research was conducted in the absence of any commercial or financial relationships that could be construed as a potential conflict of interest.

## Publisher’s note

All claims expressed in this article are solely those of the authors and do not necessarily represent those of their affiliated organizations, or those of the publisher, the editors and the reviewers. Any product that may be evaluated in this article, or claim that may be made by its manufacturer, is not guaranteed or endorsed by the publisher.

## Footnotes

^1^Mental disorders refer to a wide range of mental problems that can affect a person’s thinking, emotions and behavior. Examples include depression, anxiety, schizophrenia, bipolar affective disorder, PTSD and many others. These disorders are often caused by a combination of genetic, biological, environmental and psychological factors. Behavioral disorders are a type of mental disorder that affects a person’s behavior. They can be defined as patterns of behavior that are socially unacceptable, interfere with a person’s daily functioning, or both. Examples include ADHD, oppositional defiant disorder, chronic disorders, impulsivity, aggression and others. These disorders often begin in childhood or early adolescence, but can also occur in adults.
